# Division of labor between IRF1 and IRF2 in regulating different stages of transcriptional activation in cellular antiviral activities

**DOI:** 10.1186/s13578-015-0007-0

**Published:** 2015-04-18

**Authors:** Gang Ren, Kairong Cui, Zhiying Zhang, Keji Zhao

**Affiliations:** College of Animal Science and Technology, Northwest A&F University, Yangling, Shaanxi 712100 PR China; Systems Biology Center, Division of Intramural Research, National Heart, Lung and Blood Institute, National Institutes of Health, Bethesda, MD 20892 USA

**Keywords:** IRF1, IRF2, BRG1, ChIP, Transcription, Antiviral activities

## Abstract

**Background:**

Cellular antiviral activities are critically controlled by transcriptional activation of interferon-inducible genes, involving interferon regulatory factors (IRFs). Previous data suggested that IRF1 is an activator and IRF2 is a repressor, which functionally antagonize each other in transcriptional regulation. However, it is not clear how these two factors function to regulate cellular antiviral activities.

**Results:**

We show that IRF2 is critically required for the induction of the TLR3 and other interferon-inducible genes in a chromatin environment. While both IRF1 and IRF2 directly interact with the BAF chromatin remodeling complex, IRF2 is associated with the TLR3 promoter in the unstimulated state and IRF1 binding to the promoter is strongly induced by stimulation with interferon, suggesting that these two factors may function at different stages of gene induction in the recruitment of the BAF complex. IRF2 acts to maintain the basal level expression, an open chromatin structure, and active histone modification marks (H3K9, K14 acetylation and H3K4 tri-methylation) of the TLR3 promoter in the unstimulated state, while IRF1 serves to rapidly activate the promoter upon stimulation.

**Conclusions:**

IRF1 and IRF2 of the IRF family of transcription factors play distinct roles in cellular response to viral infection. IRF2 binds to TLR3 and other IFN-inducible gene promoters and maintains an active chromatin structure in the unstimulated state, which is required for their induction, while IRF1 binding to these promoters activates their transcription upon viral infection. Thus, the division of labor between the IRF transcription factor family members plays a pivotal role in coordinating the transcriptional activation in the cellular antiviral response.

## Background

Genome analyses indicate that human genome contains only about twice the number of genes than the simple nematode worm, C. *elegans*, has [1-3]. It has been hypothesized that the more complexity of human compare to the worm is caused by the diversity of transcriptional regulatory DNA elements and transcription cofactor complexes [4]. Gene duplication in human genome results in the existence of multiple family members of transcription factors, which usually have highly conserved DNA-binding domains and recognize the same DNA sequence. How do the different family members contribute to the complexity and accuracy of their target genes regulation? To provide insights to this question, we decided to study the transcriptional regulation of toll-like receptor 3 (TLR3) by the interferon regulatory factors IRF1 and IRF2 [5]. IRF1 and IRF2 belong to the nine-member IRF family with highly homologous N-terminal DNA-binding domains [6-8]. Previous studies suggested that IRF1 is an activator and IRF2 is a repressor of transcription; and they function antagonistically by recognizing the same DNA motif [9].

TLR3 is a critical regulator of cellular antiviral activities [10]. It recognizes double-stranded (ds) RNA, which is an intermediate of viral replication, and transmits signals to induce the key cytokines of the cellular antiviral system, the type I interferons IFN-α/β [6]. The expression of IFN-α/β is activated by NF-κB and TLR3. IFN-α/β signal through their cell surface receptors to activate and translocate the trimeric ISGF3 complex consisting p48, STAT1, and STAT2 to the nucleus, which is required for the activation of hundreds of target genes [11]. Both the basal and induced levels of expression of the interferon-inducible genes are critical for the innate and activated cellular antiviral activities [12]. Thus understanding the transcriptional regulation of TLR3 will provide insights to the cellular antiviral activities. In this study we present data to show the differential function of IRF1 and IRF2: IRF2 is associated with TLR3 and other IFN-inducible gene promoters in unstimulated states and potentiates their induction in response to viral infection by maintaining an active chromatin structure while IRF1 activates transcription of these genes in response to viral infection.

## Results and discussion

**Figure 1:**
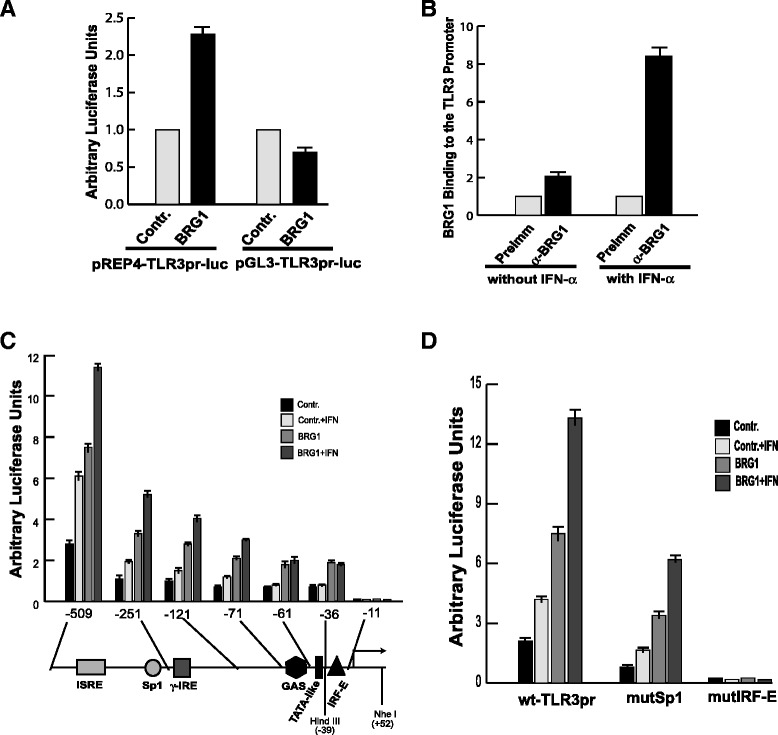
IRF-binding site mediates the BAF complex activity at the TLR3 promoter. **A** The TLR3 promoter is regulated by BAF complex in a chromatin-dependent manner. SW-13 cells were transfected with the pREP4 or pGL3 reporter vectors in the absence or presence of a BRG1 expression vector for 48 hours. The luciferase activity was analyzed using a dual-luciferase assay kit from Promega. Error bars indicate the range of three independent experiments. **B** The BAF complex is associated constitutively with the TLR3 promoter. Chromatin was prepared by sonication from HeLa cells treated with IFN-α for 12 hours prior to formaldehyde cross-linking. DNA purified from immunoprecipitate with antibody against BRG1 or pre-immune serum was analyzed with primers covering the TLR3 promoter using quantitative-PCR. **C** Deletion analysis of the TLR3 promoter. The TLR3 promoter was deleted from 5′ end and analyzed similarly as in panel A. IFN indicates the cells were treated with 500 units/ml if IFN-α for 12 hours before harvesting for analyzing the luciferase activity. The potential transcription factor binding sites in the promoter region indicated below the panel. The numbers indicate that the position of deletion and are relative to the transcription initiation site. The Hind III and Nhe I sites used in the restriction enzyme accessibility assays (Figure 4A) are also indicated. **D** The IRF-binding site is essential for the TLR3 activity. The Sp1 and IRF-E sequences in the TLR3 promoter were point-mutated respectively and analyzed as in panel C. wt-TLR3pr: the wild type TLR3 promoter in pREP4-luc reporter vector; MutSp1: the Sp1 binding site in the TLR3 promoter was point-mutated; mutIRF-E: the IRF binding site in the TLR3 promoter was point-mutated.

To gain more insight into how the innate and induced antiviral activities are controlled on gene expression levels, we studied the molecular mechanisms that control the expression of the TLR3 gene that is induced by viral infection. Both the basal level expression and induction of TLR3 require the chromatin remodeling activity of the SWI/SNF-like BAF complexes, since the knockdown of an essential subunit of the complex, BAF47, severely inhibited its expression [12-15]. To confirm that the TLR3 promoter is a direct target site of the BAF complex, the promoter region [16] was cloned into pGL3, which does not form regular chromatin structure, or pREP4 reporter vector, which replicates and forms regular chromatin structure when transiently transfected into cells [17]. Co-transfection with BRG1 expression construct into SW-13 cells, a BRG1-deficient cell line, significantly activated the promoter with pREP4 vector but not with the pGL3 vector (Figure 1A), suggesting that the BAF complex regulates the TLR3 promoter in a chromatin-dependent manner. Chromatin immunoprecipitation (ChIP) assays revealed that anti-BRG1 antibody pulled down the endogenous TLR3 promoter without IFN-α treatment (Figure 1B), indicating the BAF complex is constitutively associated with the promoter. IFN-α stimulation enhanced BRG1 binding to the promoter as shown by quantitative-PCR (q-PCR) analysis (Figure 1B). These results indicate that the TLR3 promoter is a direct target site of the BAF complex.

To identify the DNA elements that mediate the BAF complex activity, 5′-deletion analysis of the TLR3 promoter was performed. The 509 bp DNA fragment of promoter was responsive to IFN-α stimulation and to the BRG1 expression (Figure 1C). Both together can activated the promoter further. Deletion to −251 reduced the activity of the promoter. However, the responsiveness to IFN-α and BRG1 remained, even though an apparent ISRE was deleted. Deletion to −11, which removed an IRF-E sequence, completely abolished the activity of the promoter. These data suggest that the Sp1 binding site contributes to and the IRF-E plays an essential role in mediating the activity of the BAF complex. Consistent with this, the point mutation of the Sp1 binding site decreased the promoter activity, and point mutation of the IRF-E site completely abolished the activity of the promoter (Figure 1D). These results are consistent with the observation that Sp1 stabilizes the BAF complex binding to target promoters [18].

**Figure 2:**
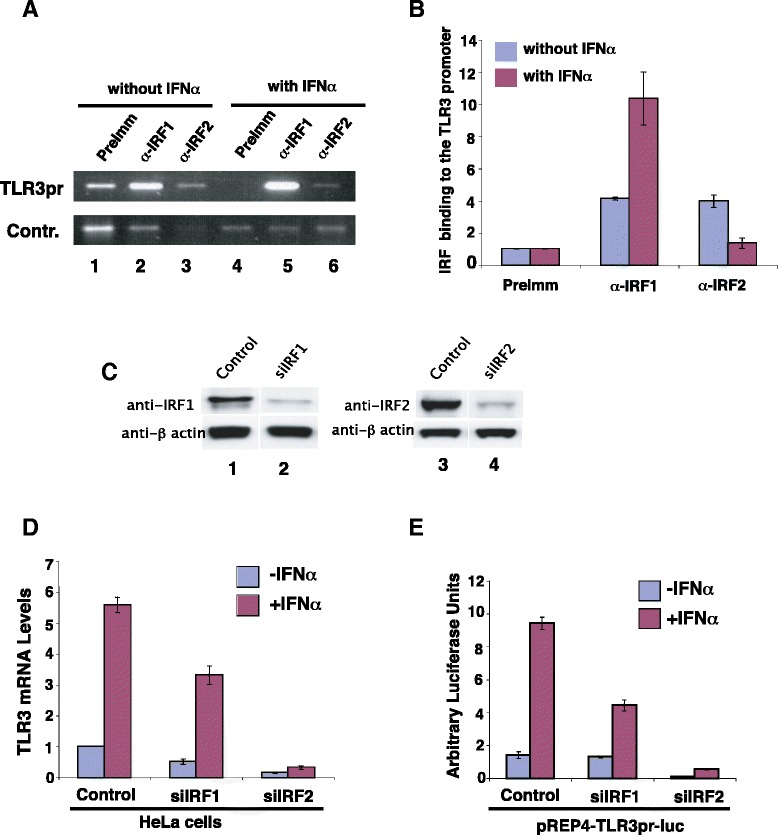
IRF1 and IRF2 differentially regulate the basal and induced expression of the TLR3 gene. **A** IRF1 and IRF2 binding to the TLR3 promoter in the basal and stimulated states. Chromatin was prepared from HeLa cells with or without IFN-α treatment for 12 hours as in Figure 1B and subjected to immunoprecipitation using anti-IRF1 and anti-IRF2 antibodies. The ChIP DNA was analyzed using specific primers for the TLR3 promoter or for the control sequence of the β globin gene. **B** The ChIP DNA in panel A was quantified using real-time PCR analysis. **C** IRF1 and IRF2 were knocked down using small interference RNA constructs. HeLa cells were transfected with pREP4-siIRF1 or pREP4-siIRF2. The cells were harvested after selection with puromycin (1 ug/ml) for 2 days and analyzed by Western blotting. The control is a small interference RNA construct that failed to knock down IRF1. β actin was used as a protein loading control. **D** Knocking-down IRF1 and IRF2 inhibits the expression of the TLR3 gene. HeLa cells were transfected with a control, siIRF1, or siIRF2 and selected with puromycin for two days. Following stimulation with IFN-α for 12 hours, total RNAs were isolated and the TLR3 mRNA levels were determined using q-PCR. **E** Knocking-down IRF1 and IRF2 inhibits the TLR3 promoter activity. pREP4-TLR3pr-luc was co-transfected into HeLa cells with a control, or siIRF1, or siIRF2 construct for 48 hours. Following stimulation with IFN-α for 12 hours, the cells were harvested and luciferase activity determined as in Figure 1A.

IRF-E sequence is the binding site for IRFs [19]. ChIP assays showed that both the IRF1 and IRF2 antibodies enriched the TLR3 promoter sequence relative to the control in non-stimulated cells (Figure 2A and B). Interestingly, IFN-α treatment strongly enhanced IRF1 binding but inhibited IRF2 binding (Figure 2A and B). No binding was detected for other IRFs (data not shown). These data suggest that IRF1 and IRF2 may regulate both the basal and induced expression of the TLR3 gene. To confirm this, both genes were knocked down by small interference RNA (siRNA) constructs (Figure 2C). When IRF1 was knocked down, the induction of the TLR3 gene (Figure 2D) and its promoter activity (Figure 2E) were significantly reduced. The basal level expression was only slightly decreased by siIRF1. Surprisingly, knocking down IRF2 resulted in a dramatic reduction of both the basal and induced levels of the TLR3 gene expression and also its promoter activity (Figure 2D and E). These data reveal that while IRF1 is critical for the induction of the TLR3 gene, IRF2 binding to the TLR3 promoter plays an essential role in both the basal and induced expression of the TLR3 gene in response to interferon stimulation.

Why do the cells take the trouble to make two proteins binding to the same site? We hypothesize that the TLR3 gene needs to be expressed at low level in the absence of viral infection and needs to be rapidly induced to high level in the presence of viral infection. Furthermore, the chromatin structure at the promoter should be prepared for rapid activation in response to viral infection. Based on the *in vivo* binding and knock-down results (Figure 2), we hypothesize that IRF2 may be the factor to prepare the chromatin structure and direct low level expression, and IRF1 may be the factor to direct highly induced expression.

**Figure 3:**
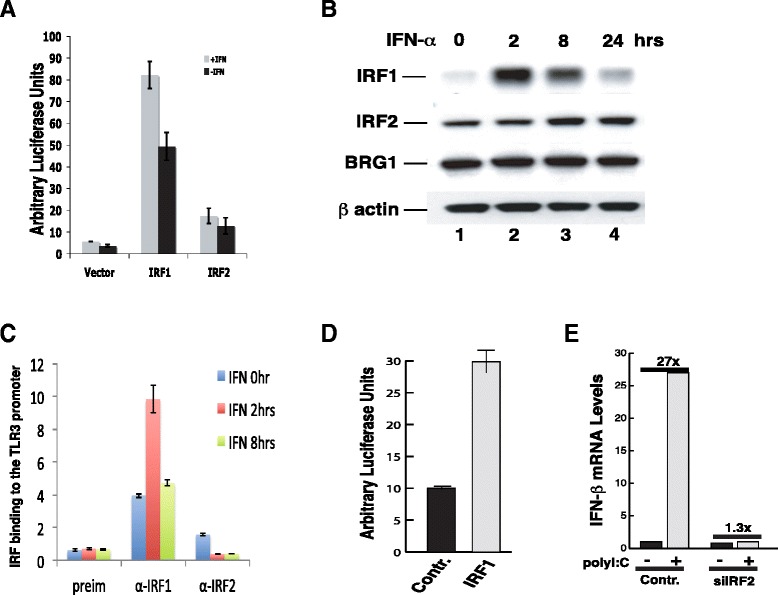
The binding of IRF1 and IRF2 to the TLR3 promoter is regulated by their protein levels. **A** IRF1 is a strong activator whereas IRF2 is a weak activator. A luciferase reporter construct containing five GAL4 DNA binding sites was co-transfected to HeLa cells with a construct expressing either the GAL4 DNA binding domain-IRF1 fusion or the GAL4 DNA binding domain-IRF2 fusion protein for 48 hours, followed by treatment with IFN-α for 12 hours. Luciferase activity was measured as in Figure 1A. **B** IRF2 is expressed constitutively and IRF1 is rapidly induced by IFN-α treatment. HeLa cells were treated with IFN-α for various times as indicated above the panel. The cells were harvested and analyzed for the protein levels of IRF1, IRF2, and BRG1 using Western blotting. β actin was used as a loading control. **C** IRF1 and IRF2 binding to the TLR3 promoter. HeLa cells were treated with IFN-α for various times as indicated within the panel. ChIP assays were performed and analyzed using q-PCR as in Figure 2A & B. **D** Over-expression of IRF1 activated TLR3 promoter. pGL3-TLR3pr-luc was co-transfected with a control vector or IRF1 expression vector, pcDNA4-IRF1 into HeLa cells for 48 hours as indicated below the panel. The luciferase activity was determined as in Figure 1A. **E** IRF2 is required for induction of IFN-β by polyI/polyC. HeLa cells transfected with the control or siIRF2 constructs were selected in puromycin for two days. Following treatment with 20 μg/ml of polyI/polyC for 6 hours, the total RNAs were isolated and analyzed for the presence of IFN-β mRNA by real-time PCR.

IRF2 has been considered as a transcriptional repressor or activator [5]. To test our hypothesis above, first, we decided to distinguish if IRF2 is a repressor or weak activator. When fused to the GAL4 DNA binding domain, IRF1 efficiently activated a promoter containing five GAL4 DNA binding sites, whereas IRF2 showed only low activity (Figure 3A). These results indicate that IRF1 is a strong activator and IRF2 is a very weak activator. Second, we addressed how the IRF1 and IRF2 binding to the promoter were controlled. Western blotting indicated that IRF1 protein was highly induced by IFN-α treatment, with the highest protein level detected after 2 hours of stimulation (Figure 3B). The IRF1 level decreased significantly after 8 hours and went back to basal level at 24 hours post stimulation. In contrast, the IRF2 protein level was relatively stable (Figure 3B). Remarkably, IRF1 binding to the TLR3 promoter paralleled with its protein level in the cells. The highest binding level was detected after 2 hours of IFN-α treatment and the binding level decreased to almost basal level after 8 hours of stimulation (Figure 3C). These data argue that the binding levels of IRF1 and IRF2 at the TLR3 promoter are controlled by their relative protein levels in the cells. If this true, artificially increasing IRF1 level in the cells would be able to compete with IRF2 and activate the TLR3 promoter. Indeed, over-expression of IRF1 efficiently activated the TLR3 promoter (Figure 3D), consistent with its ability to compete with IRF2 to bind to the promoter and its activity as a strong activator.

Activation of the TLR3 gene by viral infection or polyI/polyC leads to the induction of the IFN-β gene, which is mediated by NF-κB and TLR3 [10]. Our data that IRF2 is required for the basal and induced levels of TLR3 expression suggest that knocking down IRF2 may cripple the cellular response to polyI/polyC. To confirm this, HeLa cells, which were either transfected with a control vector or siIRF2, were treated with polyI/polyC. The induced expression of the IFN-β gene was abolished by knocking down IRF2 (Figure 3E), suggesting that IRF2 is a key molecule that controls the cellular antiviral activities.

**Figure 4:**
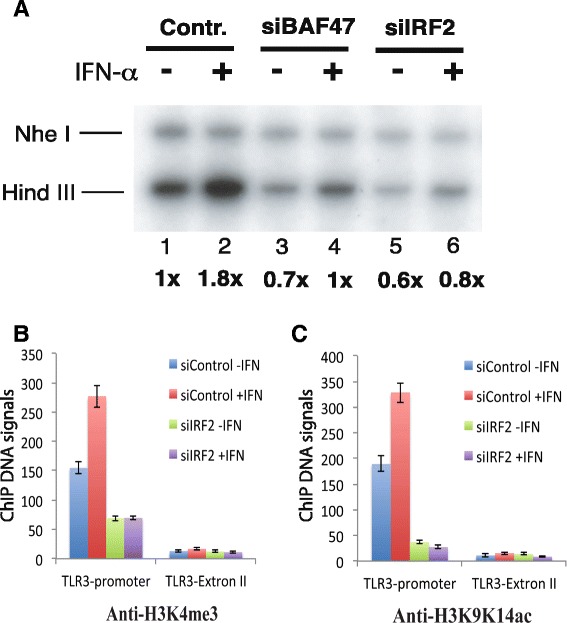
IRF2 is required for chromatin accessibility and active histone modifications at the TLR3 promoter. **A** IRF2 and BAF47 are required for the chromatin accessibility at the TLR3 promoter. HeLa cells transfected with the control or small interference RNA constructs as indicated above the panel were selected with puromycin for three days. Following treatment with IFN-α for 12 hours, HeLa nuclei were isolated and briefly digested with Hind III for 10 minutes. The purified genomic DNA was digested to completion with Nhe I and analyzed by LM-PCR using primers specific for the TLR3 promoter. The data were quantified using phosphoimager analysis. The intensity of the Hind III bands were normalized by that of the Nhe I bands and indicated below the panel. The positions of Hind III and Nhe I sites were indicated in Figure 1C. **B** IRF2 is required for H3K4me3 modification at the TLR3 promoter in both unstimulated and stimulated states. HeLa cells transfected with the control or small interference RNA construct targeting IRF2 were selected with puromycin for three days. Following treatment with IFN-α for 12 hours, chromatin fractions were prepared and immunoprecipitated with H3K4me3 antibodies. The resulting DNA samples were analyzed using q-PCR with primers for either the TLR3 promoter or exon II regions. **C** IRF2 is required for H3K9ac/K14ac modification at the TLR3 promoter in both unstimulated and stimulated states. HeLa cells transfected with the control or small interference RNA construct targeting IRF2 were selected with puromycin for three days. Following treatment with IFN-α for 12 hours, chromatin fractions were prepared and immunoprecipitated with H3K9ac/K14ac antibodies. The resulting DNA samples were analyzed using q-PCR with primers for either the TLR3 promoter or exon II regions.

To investigate how IRF2 regulates the induction of TLR3, we examined the accessibility of the TLR3 promoter to restriction enzyme Hind III, which has a recognition site −39 bp upstream of its TSS. We found that interferon-α treatment increased the accessibility of the Hind III site, while no changes were detected at the Nhe I site which is located at +52, downstream of the TSS (Figure 4A, lanes 1 and 2). knocking down of BAF47, a key subunit of the BAF chromatin remodeling complex, resulted in decreased accessibility of the Hind III site as compared to the control Nhe I site (Figure 4A, lanes 3 and 4), consistent with our previous observation that the BAF complex is involved in the induction of IFN-inducible genes [12,20]. Interestingly, knocking down of IRF2 also compromised the accessibility of the Hind III site (Figure 4A, lanes 5 and 6). These results indicate a critical role of IRF2 in maintaining an open chromatin structure at the TLR3 promoter in both non-stimulated basal and stimulated states.

Next we investigated whether IRF2 binding impacts histone modifications. Histone H3 is subject to extensive modifications including acetylation and methylation, which is correlation with transcriptional activation or repression [21]. To test whether IRF2 facilitates histone modifications at the TLR3 promoter, we measured H3K4me3 and H3K9/K14ac using ChIP assays. Our data revealed that these modifications at the promoter region were dramatically decreased by knocking down IRF2, while no significant changes were detected at the exon II region of the TLR3 gene (Figure 4B, C). These data confirmed our hypothesis that IRF2 is essential for keeping an open chromatin structure at the TLR3 promoter.

**Figure 5:**
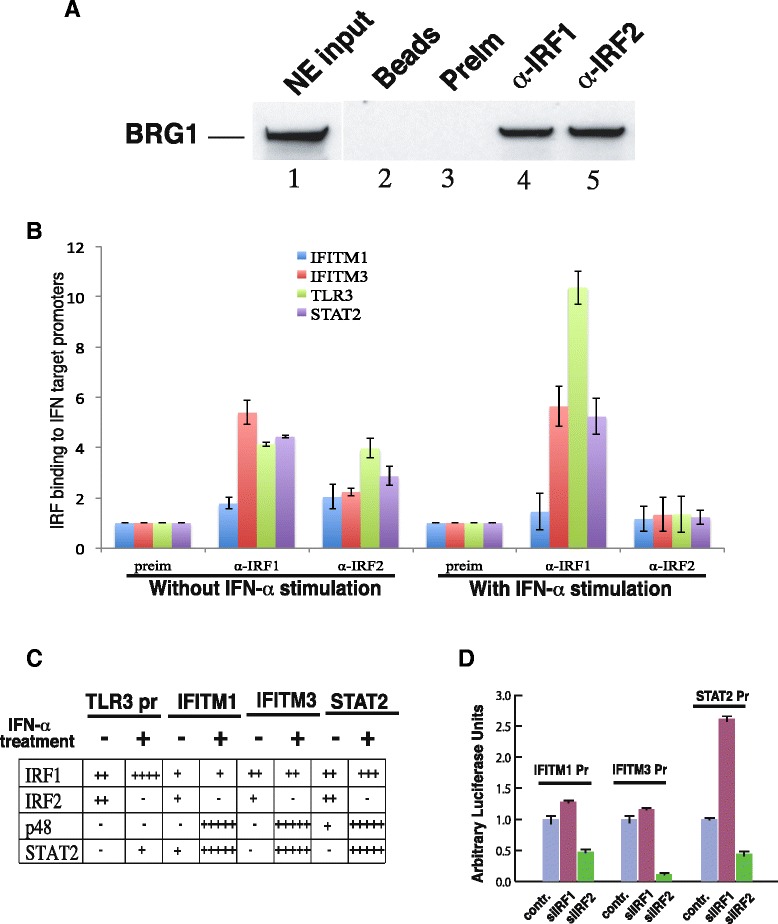
IRF2 recruits the BAF complex to other IFN target promoters. **A** IRF1 and IRF2 interact with BAF complex in nuclear extracts. Nuclear extracts prepared from HeLa cells were immunoprecipitated with the indicated antibodies. The immunoprecipitates were resolved by SDS-PAGE and blotted with BRG1 antibodies. Two percents of the nuclear extracts used for IP was loaded in lane 1 as input. **B** Both IRF1 and IRF2 bind to IFN target promoter without IFN-α stimulation while IRF2 dissociates from IFN target promoters after IFN-α stimulation. ChIP using chromatin from HeLa cells with or without IFN-α stimulation was performed and analyzed by q-PCR as in Figure 2B. **C** Interplay of IRFs and ISGF3 at IFN target promoters. ChIP assays using HeLa cells with or without stimulation with IFN-α for 12 hours were performed. The binding of IRF1, IRF2, p48, and STAT2 to promoters as indicated was analyzed by q-PCR and the data were summarized. **D** IRF2 but not IRF1 is required for the basal level activity of IFN target promoters. Different promoter constructs in pREP4-luc vector as indicated above the columns were co-transfected with the RNA constructs indicated below the columns into HeLa cells for 48 hours. The luciferase activity was measured as in Figure 1A.

The data that knocking-down IRF2 reduced the chromatin accessibility of the TLR3 promoter to a similar extent as knocking-down BAF47 suggest that IRF2 may be responsible for the recruitment of the BAF complex to the promoter in non-stimulated cells. Thus, we performed co-immunoprecipitation assays to test the direct interaction between IRF2 and the BAF complex. Our data revealed that BRG1 was co-immunoprecipitated from the nuclear extracts using both anti-IRF1 and anti-IRF2 antibodies (Figure 5A). Addition of ethidium bromide did not inhibit the co-immunoprecipitation, indicating that the interaction was not mediated by DNA. These data suggest that both IRF1 and IRF2 are capable of recruiting the BAF complex to the TLR3 promoter to maintain an open chromatin structure through protein-protein interactions.

IRF1/2 are known to recognize ISRE [22], which mediates the induction of IFN-α target genes by the ISGF3 complex. Since the BAF complex regulates most of the IFN-α target genes [12], IRF2 may serve as a general recruiter of the BAF complex to the IFN-α target genes. To test this idea, we analyzed IRF1 and IRF2 binding at several IFN-α inducible genes by ChIP assays. As shown in Figure 5B and C, both IRF1 and IRF2 bound to the promoters of TLR3, IFITM1, IFITM3, and STAT2. Interestingly, IRF2 disappeared from all of these promoters after IFN-α treatment. We assume that IRF2 was replaced by the ISGF3 complex at the IFITM1, IFITM3, and STAT2 promoters as suggested by the ChIP data summarized in Figure 5D. Similarly to the induction of TLR3, knocking down of IRF2 also significantly inhibited the expression of IFITM1, IFITM3, and STAT2 (Figure 5E). Therefore, these data define a general role of IRF2 to potentiate the induction of the IFN-α target genes in response to viral infection.

Recent studies suggested that epigenetic mechanisms play roles in controlling the function of IRF1 and IRF2 [23]. In particular, histone acetyltransferases can directly enhance their transcriptional activity by modifying nucleosome or IRFs themselves [24,25]. Our previous studies suggested that the ATP-remodeling BAF complexes are required for maintaining the basal and induced expression of IFN-inducible genes [12]. However, it is not clear what are the roles of the constitutively expressed IRFs in this process. Although IRF2 has been considered a transcriptional repressor and an antagonist of IRF1 [5,26], it has been difficult to explain certain phenotypes in mice associated with disruption of IRF1 and IRF2 [27,28]. In this study, our data argue against the notion that IRF2 is a repressor and thus antagonizes the activity of IRF1. Instead, we demonstrate that IRF1 is a strong transcription activator and IRF2 is a weak activator, which act at different stages of TLR3 activation. IRF2 serves two roles at the TLR3 promoter: 1) to recruits the BAF complex to prepare an open chromatin structure for rapid activation upon viral infection; 2) to maintain a basal level expression of the gene for the innate antiviral activity. IRF1 serves to rapidly activate the promoter by replacing IRF2 upon induction. However, if the chromatin structure at the promoter was not prepared, induction completely failed. The functional difference between IRF1 and IRF2 may arise from two mechanisms. One is the control of their expression: IRF2 is constitutively expressed while IRF1 is inducible by viral infection or interferon treatment, which results in different binding patterns during different stages of cellular antiviral activity. The other may reflect the differences of their activation domains: although IRF1’s activation domain can potently activate transcription, our data argue that only the activation domain of IRF2 can act as a pioneering factor to prepare chromatin for rapid transcription induction in response to stimulation. Therefore, our data in this report demonstrate that different members in a transcription factor family are made to meet different requirements for elaborate transcriptional regulation. The vertebrate organisms gain an extra level of transcriptional control by duplication of gene families.

## Materials and methods

### Constructs and antibodies

pREP4-TLR3pr-luc, and pGL3-TLR3pr-luc were constructed by cloning the PCR-amplified 509 bp fragment of the TLR3 promoter (from −509 to +1) into appropriate restriction sites in the pREP4-luc [17] and pGL3 vectors. The promoter deletion reporter constructs were generated by cloning the corresponding PCR fragments in the pREP4-luc vector. The reporter constructs with point mutations of the Sp1 and IRF-E motifs were generated from the 509-bp promoter construct using a Stratagene mutagenesis kit. The shRNA constructs for IRF1 and IRF2 were generated by inserting the corresponding cDNA sequences into the pREP4-puro vector. pREP4-U6-shBAF47 construct was described previously [12].

The oligonucleotides for Sp1 and IRF-E site mutations are listed below:

The Oligonucleotides used for shRNA constructs are listed below:

### Antibodies used in this study are from the following sources

Anti-IRF1(sc-497X; Santa Cruz), anti-IRF2(sc-498X; Santa Cruz), anti-TLR3(H-125; Santa Cruz), anti-BRG1(sc-17796X; Santa Cruz), anti-hBRM(sc-6450; Santa Cruz), anti-BAF47(home made see paper), anti-p48(sc-496; Santa Cruz), anti-Stat1(06–502; upstate), anti-Stat2(sc-346-G; Santa Cruz), anti-PloII (sc-899X; Santa Cruz), anti-CBP(ab10489; abcam), anti-H3K4me3(17–614; Millipore), anti-H3k9ac/14 ac (ab4441;abcam), anti-H3(ab1791-100; abcam).

### Cell culture and transfection

SW-13 cells and HeLa cells were maintained in DMEM supplemented with 10% fatal calf serum and 1% penicillin-streptomycin. The THP-1 cells were maintained in ATCC-formulated RPMI-1640 Medium (Catalog No. 30–2001) supplemented with 10% fatal calf serum and 1% penicillin-streptomycin. Transfections of SW-13, HeLa cells, and THP-1 cells were performed using Superfect (Qiagen) as instructed by manufacture. The cells were selected in 1 μg/ml puromycin for 48 hours before stimulation with 500 Units/ml IFN-α for 12 hours for chromatin immunoprecipitation and TLR3 gene expression analysis.

### RT-PCR analysis

TaqMan® Gene Expression Assays for TLR3 and IFN-β gene were ordered from ABI (Cat. # Hs01551078_m1; Cat. # Hs01077958_s1). RT-PCR analyses were performed as described previously [17], by using total RNAs isolated from HeLa, SW-13, and THP-1 cells.

### ChIP

The chromatin immunoprecipitation assays were formed as described [18]. For the LTR3 gene promoter region: Forward primer: 5′-*CCGCCCACATCAAATGGT*-3′, Reverse primer: 5′-*GAAAGGGTCACAGATTTAGCAACA*-3′, Probe: 5′-*CCCACTTTCAACTTTAG*-3′ were used. For the LTR3 gene extron II region: Forward primer *5′-GTGCATCCTCCACCACCAA-3′,* Reverse primer *5′-TCGGGTACCTGAGTCAACTTCA-3′,* and Probe: *5′-TGCACTGTTAGCCATGAAGTTGCTGACTG-3′* were used. For β-globin gene locus:

- Forward primer: 5′-*ACAGTGTGGCGATTCCTCAAG*-3′, Reverse primer: 5′-*GTAATGGGATTGCTGGGTCAA*-3′, Probe: 5′-*ATCTAGAACCAGAAATACTG*-3′ were used.
